# Green spaces and the impact on cognitive frailty: a scoping review

**DOI:** 10.3389/fpubh.2023.1278542

**Published:** 2024-01-12

**Authors:** Sally Fowler Davis, Charlotte Benkowitz, Lucie Nield, Chris Dayson

**Affiliations:** ^1^School of Allied Health and Social Care, Anglia Ruskin University, Cambridge, United Kingdom; ^2^Advanced Wellbeing Research Centre, Sheffield Hallam University, Sheffield, United Kingdom

**Keywords:** green spaces exposure, older people, cognitive frailty, scoping review, urban—rural

## Abstract

Some literature indicates that contact with green spaces can benefit health and wellbeing, but it is unclear whether this is protective of cognitive health in older people. Using Arskey and O’Malley’s framework the aim was to investigate ageing, cognitive frailty and the effects of green access including any causality. The evidence was somewhat inconsistent but suggestive for a beneficial role of green space exposure on cognitive functions. Results suggested that globally, the poorer urban environments are high risk for older people’s mental health and these places often lack parks and green spaces. There is evidence that the level of activity and social participation may be greater with access to green spaces and therefore reduces health risks. Green spaces seem to have a role in preventing cognitive frailty, especially for more vulnerable older populations living in poorer urban environments.

## Introduction

According to the World Health Organisation, the definition of public health is “the art and science of preventing disease, prolonging life and promoting health through the organised efforts of society” (1). Public health therefore extends beyond the provision of health services and includes the assessment and evaluation of neighbourhoods, communities and environments that may be pivotal to physically active ageing. Health impact assessments (HIA) are undertaken by public health organisations to predict the cost, effectiveness and scalability of policy to improve population health (2). A critique of available primary data relating to the topic is particularly important for the HIA and is used to evidence any strategic urban planning and investment associated with healthy ageing with older populations.

Cognitive frailty (*CF*) is the coexistence of both physical frailty and cognitive impairment in older persons without dementia (3). The decline in cognition results in higher risk of falls and physical deconditioning and visa versa (4). The incidence of frailty is common in community dwelling older adults (5) and as a physical syndrome is based on three or more of the following criteria: unintentional weight loss, self-reported exhaustion, weakness and slow or low physical activity (6). *CF* is less well recognised clinically although distinct from mild cognitive impairment because *CF* can be reduced, and functional health improved, with community-based interventions (7). With the rapid increase of the ageing population, health promotion and prevention strategies are essential and there are opportunities for early interventions to reverse the functional impacts of cognitive frailty (8).

The term ‘green space’ is used widely in the academic literature pertaining to the role of the natural environment in determining physical and cognitive health. Green spaces are ‘any vegetated areas of land or water within, or adjoining an urban area’, including parks and gardens, allotments, community gardens and city farms, cemeteries and churchyards among others (9). The natural environment presents both threats to health (e.g., air pollution or biological hazards) but can be a setting through which to promote good health. For example, in 2017 an article in the Lancet identified providing *‘green space and subsidised sport and recreation facilities’* as a contributory action for addressing health inequalities (10) and equitable access to urban green spaces is one of the contributory Sustainable Development Goals (11).

A large body of international literature points to a positive association between good health and wellbeing and time spent in nature (12–15) with benefits including lower body fat (16), fewer cardiovascular and respiratory problems (17) and obesity management (18). Exposure to natural environments appears to increase wellbeing via stress reduction (19), attention restoration (20) and biophilia (21), reflecting a wider argument that humans are inherently drawn to nature and are less well adapted to modern urban environments (22). Benefits are greatest for those at risk of ill-health, including individuals with dementia and cognitive loss (23–26) and those with severe mental ill-health conditions (27, 28) where three-quarters of relevant studies reported a positive association between brain health and green space exposure (24).

This scoping review reports on a selected literature that analyses the association between cognition, ageing and green space to identify possible effects of access to natural environments as a mediator to the risk of *CF.* Given the incidence of *CF* (between 10 and 12% of the over 65 year old, community dwelling older population) (3), the study is intended to indicate the opportunities for wider public health research into urban planning and environmental assets in communities.

## Methods

### Literature searching

This review was undertaken as part of a wider project to assess environmental risks in relation to *CF* in older people and based on a search strategy that included air quality, social environment *and* green spaces. Due to the scope of the literature this paper reports on the data relating to green spaces and *CF* in older adults. The scoping review was conducted using the framework guidelines by Arksey and O’Malley (29) using Scopus to search. Subsequent selection and categorisation was undertaken in a systematic way. Scopus was selected because it has global coverage of scientific journals and covers all scientific topic areas. Using the six-stage process (29) the searches were derived through discussion and data collation and included peer reviewed publications in English and excluded specific reference to dementia/Alzheimer’s disease and frailty as a clinical description. Physiological and physical frailty was excluded to (a) limit search results to *CF* as defined and to (b) identify the literature that identified environmental factors including green spaces.

**Figure 1:**
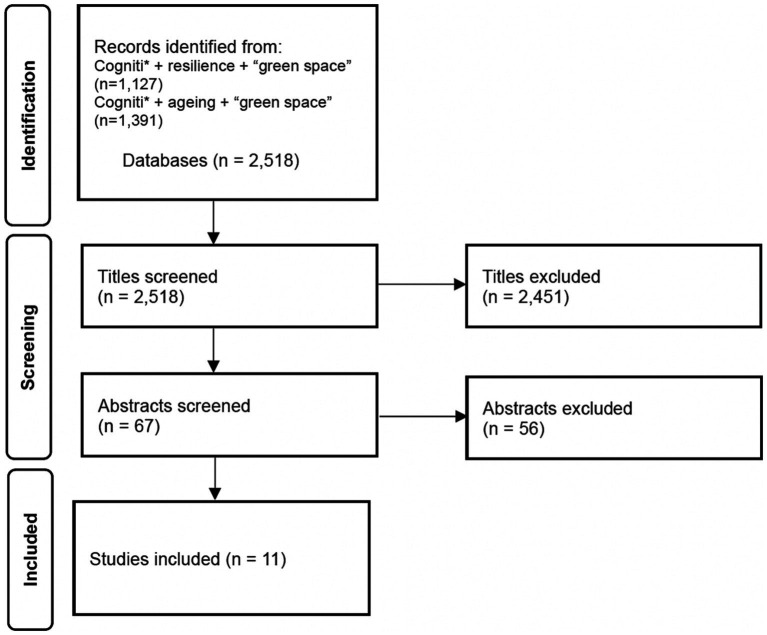
PRISMA (31).

Scoping reviews enable rapid and pragmatic assessment of the data (30). Given the limited literature exploring *CF* in relation to environmental risks, a scoping review was an appropriate method, providing an indication of the associations between ageing, cognition and resilience. The search terms included cogniti* AND resilience and “green space” as well as cogniti* AND ageing AND “green space and ‘older adult’ in the title. For study selection, duplicates were removed followed by a title screening which allowed for the removal of any books, reviews and commentaries. The rigorous exclusion of studies on dementia and physical frailty resulted in a more targeted literature on *CF* (see PRISMA Figure 1). Abstract screening enabled further refinement; articles were included if the study indicated that the impact of green space exposure on cognition was explored.

### Data synthesis

A Microsoft Excel sheet was used for data extraction, based on the conceptual themes identified alongside research and publication details including; the sample size, participants age range, the gender split, participants’ educational attainment and social or financial status. Furthermore, information on how green space and cognition were measured was included, providing a baseline for the assessment of the value of the overall findings (but without undertaking critical appraisal of the literature (29)). The data was then synthesised, using the data extraction tool, by comparing the findings, relative to the themes. The data reporting is linked to the conceptual framework, using literature to identify how the data across studies makes the association and indicates the type of data and availability of research (32).

## Results

### Included papers

Following searches, screening and data selection based on the conceptual understanding of *CF* and environmental risk, 11 papers were selected for the specific domain related to green space and *CF.*

Eleven peer review publications were selected, published between 2005 and 2022 with two each from the United States, Ireland, and Taiwan, three from China (including Hong Kong), one from the United Kingdom, Canada, and Sweden. Seven studies were quantitative with one qualitative and one mixed methods. The other studies were observational cohort designs and this included a retrospective life course study (33). The total sample of older people included in the studies was 347,804 and this included a range of ethnic groups of participants who were both indigenous, i.e., Taiwanese, Chinese and Scottish or British and included racial diversity of those who self-identified as Caucasian, Aboriginal, Chinese, South East Asian, Japanese, Filipino, Dutch or German living in Canada.

The selected articles are presented along with demographic features of the older adult population groups recruited to the studies see Table 1. The selected demographic factors are associated with *CF* incidence, including gender, age and educational and economic status of the participant groups and urban setting.

**Table 1:** Included papers—demographic factors.

Publication year	Authors	Title	Country of origin	Sample size	Mean age	Gender (female/male %)	Educational attainment	Economic status	Green space measure	Cognition measure	Association measure between cognition and green space
2021	Besser, L.M.; Chang, L.-C.; Evenson, K.R.; Hirsch, J.A.; Michael, Y.L.; Galvin, J.E.; Rapp, S.R.; Fitzpatrick, A.L.; Heckbert, S.R.; Kaufman, J.D.; Hughes, T.M. (34)	Associations between Neighbourhood Park Access and Longitudinal Change in Cognition in Older Adults: The Multi-Ethnic Study of Atherosclerosis	United States of America	1,733	Mean age 67.3 years (SD = 8.3 years)	53% female	9.1% of the sample had no high school degree, 15.7% of the sample had a high school degree, 30.7% of the sample had some college education but no bachelor’s degree, 44.5% of the sample had a bachelor’s degree or higher.	74.7% of the sample had a family income greater than or equal to $30,000/year.	Park access measured by the proportion of the land surrounding participants’ residence composed of parks within 0.5 miles/1 mile and straight line distance to the closest park.	Cognition was measured using the Cognitive Abilities Screening Instrument (CASI) and a Digit Symbol Coding task (DSC).	Created dichotomous measures out of the longitudinal change in the CASI and DSC scores to categories the results in a maintained/improved score or decline in score, tested associations between the continuous park measures and dichotomous cognitive variables using multivariate random intercept models.
2019	Cassarino, M.; Tuohy, I.C.; Setti, A. (35)	Sometimes Nature Does not Work: Absence of Attention Restoration in Older Adults Exposed to Environmental Scenes	United States of America	75	Mean age 68.9 years (SD = 8.3 years), range 60–95 years	56% female	80% of the sample had at least at least secondary education.	No data	Participants viewed eight images of either natural or urban environment scenes, presented on a slide show for 15 s each.	Cognition was measured using Sustained Attention to Response Task (SART).	Compared changes in attentional performance on the SART before and after exposure to natural or urban scenes (using t-tests if fulfilling the assumption of normality and Mann–Whitney tests otherwise).
2020	Chang, P.-J.; Tsou, C.-W.; Li, Y.-S. (36)	Urban-greenway factors’ influence on older adults’ psychological well-being: A case study of Taichung, Taiwan	Taiwan	769	Mean age 67.4 years, range 55–80+ years	59.9% female	25.1% of the sample had elementary school or below, 16.3% of the sample had junior high school education, 28.3% of the sample had a high school level education, 25.9% of the sample had a college degree, 4.3% of the sample master degree or above.	Participants self-reported their financial status: 7.9% as no income/poor – 45.8% as average, and 46.3% self-reported as fairly well-off or rich.	Estimated greenway environmental quality through trained professionals using the Environment Assessment of Public recreation Scale (EARPS), including facilities, seating, condition, landscaping, water, cleanliness and coverage. These were added to produce a main score.	No direct cognitive assessment tool. Rather wellbeing was measured using the PERMA model. PERMA is made up of five aspects of wellbeing (positive emotion, engagement, positive relationships, meaning and accomplishment). Participants completed 15 items (three per PERMA aspect) on an 11-point Likert scale, ranging from every time to never.	Confirmatory factor analysis and structural equation modelling were used to explore the relationships between measures.
2018	Cherrie, M.P.C.; Shortt, N.K.; Mitchell, R.J.; Taylor, A.M.; Redmond, P.; Thompson, C.W.; Starr, J.M.; Deary, I.J.; Pearce, J.R. (33)	Green space and cognitive ageing: A retrospective life course analysis in the Lothian Birth Cohort 1936	UK (Scotland)	281	Follow-up over life course at ages 70, 73, 76 and 78 years	At age 70 49.8% female, at age 78 64.9% of the analysis sample female.	No data	Childhood status assumed from father’s previous occupation sample at age 70 (age 78 in brackets): 24% (20%) professional/managerial, 64% (74%) skilled, partly skilled or unskilled, 12% (6%) NA. Adults status of participants at age 70 (78 in brackets) 54% (47%) professional/managerial, 44% (52%) skilled/partly skilled/unskilled, 2% (1%) N/A.	Park information at three different timepoints (1949, 1969, 2009) was used to determine the percentage of parks within a 1,500 m zone surrounding residence of childhood, adulthood and later adulthood periods.	Used the genetic predictor responsible for increased susceptibility to non-normative cognitive aging, the presence of APOE (apolipoprotein) e4 allele as well as the Moray House test No 12. Scores from different ages (11, 70 and 79) were used.	To explore the relationship between park availability and cognition an established method to detect the most appropriate life course model, where they build a series of linear regression models that represent the life course models under investigation and compare them to determine the most appropriate model.
2021	Finlay, J.; Esposito, M.; Li, M.; Colabianchi, N.; Zhou, H.; Judd, S.; Clarke, P. (37)	Neighbourhood active ageing infrastructure and cognitive function: A mixed-methods study of older Americans	United States of America	21,151 plus 125	Mean age 71.3 years (SD = 7.8 years)	67% female	57% of the sample had high school education or less.	No data	In interviews participants identified nearby public parks, fitness/sport amenities and walkable destinations as motivators for recreational exercise and active transit. In the quantitative part of the study generalised additive multilevel models were used to examine neighbourhood features that were identified in interviews.	Cognition was measured using a subset of the Montreal Cognitive Assessment (MoCA) including the following tests: Animal fluency, letter fluency, word list learning, world list delayed.	Fit a Gaussian generalised additive multilevel model to the sample to see how cognitive function varied among respondents living in areas with different levels of active ageing infrastructure.
2015	Finlay, J.; Franke, T.; McKay, H.; Sims-Gould, J. (38)	Therapeutic landscapes and wellbeing in later life: Impacts of blue and green spaces for older adults	United States of America	46	Range 65–85 years	No data	No data	No data	As participants mentioned in interviews.	No cognitive assessment.	Framework analysis was used to organise participants interview answers.
2022	Huang, B.; Yao, Z.; Pearce, J.R.; Feng, Z.; James Browne, A.; Pan, Z.; Liu, Y. (39)	Non-linear association between residential greenness and general health among old adults in China	China	300,442	60.78% of the sample between 60 and 69 years old, the rest older.	51.31% female	21.59% had no schooling, 68.11% had elementary or secondary education, 7.15% had senior high school education and 3.15% had a college or higher education.	The majority of the sample lived in a household without a car, had basic endowment insurance scheme and a basic medical insurance scheme, lived in houses constructed after 1990, resided in urban areas.	The census data provided geographical units that allowed the calculation of environmental variables.	No direct cognitive assessment tools. Participants self-rated their general health for an accurate assessment of their health status (good health, fair health, poor health, unable to take care of themselves).	To explore the relationship between residential greenness and self-related health, multivariate logistic models were fitted to estimate the association.
2005	Ottosson, J.; Grahn, P. (40)	Measures of restoration in geriatric care residences: The influence of nature on older people’s power of concentration, blood pressure and pulse rate	Sweden	15	Range 67–97 years (mean 86 years, median 87 years)	86.7% female	No data	No data	Participants either spent an hour indoors or outdoors.	Concentration was measured using the Necker Cube Pattern Control Test, Digit Span Forward, Digit Span Backward and the Symbol Digit Modalities Test.	Wilcoxon Rank Sum Tests were applied to test for differences in concentration before and after time spent indoors/outdoors.
2017	Wu, Y.-T.; Prina, A.M.; Jones, A.; Barnes, L.E.; Matthews, F.E.; Brayne, C.; MRC CFAS (41)	Micro-scale environment and mental health in later life: Results from the Cognitive Function and Ageing Study II (CFAS II)	Taiwan	3,590	65+ years	52% women	25.4% had 12 or more years of education, 50.6% had 10–11 years, 24% had 9 or less years.	No data	The Residential Environmental Assessment Tool (REAT) was used, a validated an observational instrument designed for measuring the quality of living environment within any given UK postcode. The REAT contains property and street level assessment by examining 28 items in four domains: physical incivilities, territorial functioning, defensible spaces and natural elements. A higher REAT score indicates a worse quality of living environment.	Participants completed the Mini-Mental State Examination (MMSE). Cognitive impairment was defined as a score of 25 of below.	Multilevel logistic regression was used to explore the association between cognition and environmental measures.
2018	Yu, R.; Wang, D.; Leung, J.; Lau, K.; Kwok, T.; Woo, J. (42)	Is Neighbourhood Green Space Associated With Less Frailty? Evidence From the Mr. and Ms. Os (Hong Kong) Study	China	3,240	Mean age 72.2 years (SD = 5.0 years)	50.8% men, 49.2% women	No data	Measured by self-report, on a ladder consisting of 10 rungs, top rung dictating people with the most satisfied income, education level, and job respect. The mean rating was 4.6 (SD = 1.8)	The percentage of green space within a 300-m buffer around the participant’s place of residence, derived from place of residence of participant at baseline. This was calculated using the normalised difference vegetation index.	Participants completed the MMSE and cognitive impairment was defined as a score of 24 of below.	Ordinal logistic regression and path analysis were used to investigate relationships between green space and the frailty transitions, (adjusting for demographics, socioeconomic status, lifestyle factors, health conditions, and baseline frailty status).
2022	Zhang, L.; Luo, Y.; Zhang, Y.; Pan, X.; Zhao, D.; Wang, Q. (43)	Green Space, Air Pollution, Weather, and Cognitive Function in Middle and Old Age in China	China	16,337	45 years and older (middle and old age); mean age 58.83 years (SD = 9.56 years)	51.4% female	26.8% none, 39.6% primary school, 33.6% middle school and above.	Mean annual household expenditure was in CNY 21,747 (SD = 19,857).	Green space coverage was calculated as the percentage of built-up areas that were covered by green vegetation. 2011 green space coverage was extracted from the 2012 City Statistical Yearbooks.	As part of a larger test battery, participants completed the MMSE.	Multilevel growth curve models were used. The mediator effects of physical activity and social engagement on the relationship between environmental factors and cognitive function were also explored.

Measures of green space and cognition are identified along with associations as reported.

The results were synthesised in relation to differences between measures of cognition and any association between urban green space and resilience/decline. Green space exposure may promote healthy cognitive ageing in older adults, but associations were seen as protective rather than curative in relation to urban living.

The impact of residing in neighbourhoods with greater availability of local parks, access to recreational amenities, and business density is associated with higher levels of cognitive function (38) as assessed using the Montreal Cognitive Assessment (MoCA) (44) although this does not confirm an association between availability of these neighbourhood resources and specifically the rate of cognitive decline. It does however suggest that successful ageing in-place is easier when supported by a facilitatory infrastructure of places to go, local amenities and municipally monitored facilities. Most studies demonstrated an association between greater park access and maintained or improved cognitive status. For example, Besser et al. (34) used the Cognitive Abilities Screening Instrument (CASI) (45) to demonstrate that greater park access had maintained or improved CASI for African Americans/Blacks but not for White participants. Similarly, Cherrie et al. (33) demonstrate that greater availability of parks in both childhood and adulthood were associated with successful cognitive ageing in later life. This longitudinal study uses linear regressions to test the association between age-standardised, change in cognitive function (Moray House Test score) (46) and noted that local provision of park space in childhood and adulthood were both important in explaining the change in cognitive function in later life. The association between childhood and adulthood park availability and change in the Moray House Test Score from age 70 to 76 was strongest for women.

For older people, living in neighbourhoods with a higher percentage of green space was associated with improvement in frailty status (including cognitive frailty) indicated using the Mini-Mental State Examination (MMSE) (47) in which cognitive impairment was defined as a score < 24. In a 2 year follow up cohort study of older people, recruiting over 3,000 participants in Hong Kong (42), participants were categorised by the amount of green space within a 300 metre ‘buffer zone’ from their primary dwelling and this was correlated with a wide range of individual characteristics including lifestyle factors. The frailty status of participants living in neighbourhoods with more than 34.1% green space at baseline were more likely to improve at the 2-year follow-up with the association between green space and frailty more significant in men. The path analysis in the study reflects that green space directly affects frailty transitions because of the positive effect of physical activity on multiple health conditions and that this was indirectly an outcome of greener environments.

Perhaps most conclusively, Zhang et al. (43) reported that greater green space coverage is associated with slower cognitive decline over a 7-year period in China. Green space, air pollution and weather condition data were extracted and merged from different sources (with limited accuracy as an acknowledged limitation) to explore the detrimental effects of the environment on ageing cognition. The analysis includes the mediating effects of physical activity, social engagement and socio-economic factors on cognitive function. Their findings show that green space coverage (along with temperature and rainfall) was positively related to cognition score (as measured by MMSE) at the baseline and associated with slower cognitive decline over the period demonstrating a, “1% increase in green coverage rate lowered the decline rate in the overall cognitive score by 0.01 points” (43)

One study showed no effect from environmental exposure on cognition. Cassarino et al. (35) found that environmental exposure had no effect on 75-year-old people for either attentional accuracy, sensitivity to visual targets or reaction times when using the Sustained Attention to Response Task (SART) (48). Greater ‘restoration’ (40) and lower ‘environmental stress’ (36) are claimed as ‘background effects’ on the general wellbeing as a self-reported outcome. The quality of neighbourhood green spaces was the main factor influencing wellbeing, when their local greenway quality was better (cleaner and better kept) then there was reported wellbeing and place attachment. Similarly, Huang et al. (39) sought to explore the association between residential greenness and older adults’ self-rated general health (SGH) in China and the impact of residential greenness to mitigate against the detrimental effects of the city environment. They concluded that higher residential greenness was positively associated with the odds of reporting good health. Older and poorer Chinese adults living in high-density environments and high levels of urbanisation may appreciate and report greater wellbeing without this having a direct effect on cognitive health. Living in a poor-quality environment was associated with nearly twice higher odds of cognitive impairment in urban conurbations (41). The assumption within the study is that the absence of green space often occurs in more deprived urban settings and exposes older people to greater risk of mental illness.

## Discussion

The evidence from this scoping review supports an earlier review relating to green space access across the lifespan, suggesting that cognitive health outcomes are inconsistent *but* suggestive for a beneficial role of green space exposure on cognitive functions (49). This study, with a focus on older adults and ageing populations indicated that there may be structural barriers to engaging with and accessing nature. For example, socioeconomic status is associated with visits to natural spaces (50, 51). Older adults are particularly susceptible to being excluded from accessing nature due to age-related changes such as reduced mobility, frailty and health deterioration (52) which leads to a “high incidence of fearfulness” reducing their motivation to venture outdoors (53).

This evidence and global recognition of the benefits to health associated with nature, have resulted in an increasing interest in how the natural environment can be used as a setting for health and care interventions and how these can be embedded in national health and environmental policies. A prominent recent UK example is the ‘Preventing and Tackling Mental Ill-health Through Green Social Prescribing Project’, which brought environmental (i.e., Defra, Natural England) and health (i.e., NHS, DHSC) policymakers together to understand how to improve the use of nature-based settings and activities to promote wellbeing and improve mental ill health (54). However, such initiatives are rare and there remains uncertainty as to how, when and where natural environments and green spaces could be best used to improve physical and cognitive health outcomes (55).

According to Attention Restoration Theory (ART) natural environments are ideal places to restore diminished attentional capacity (20). ART suggests that even short exposure to nature, as opposed to urban environments, can promote attention restoration by stimulating soft fascination and mental flexibility (20). Studies included in the review allude to the beneficial effects of nature (36) and suggest that greater neighbourhood provision of public parks from childhood through to adulthood may help to slow down the rate of cognitive decline in later life, recognising that such environmental associations are always sensitive to individual characteristics (33). It remains unclear whether particular natural environments or green spaces are more beneficial and whether this has a conclusive mitigating effect on the quality of cities and benefits to older populations (56).

Because the association between natural environments and specifically *CF* incidence remains untested, the associations reported in this scoping review are important. Growing urbanisation is a feature of the ageing demographic and there is a need to protect urban dwelling households with population-level interventions. While these studies demonstrate an association between the natural environment and cognitive health, they combine to present a strong argument for environmental interventions to ameliorate the potential risks associated with increased urbanisation for older people.

### Limitations

The evidence currently adopts a wide range of cognitive measurements which may not be comparable and similarly the definition of green space may not have been consistent in all papers. The effects of green spaces on wellbeing, particularly for those in poorer urban spaces, cannot be assumed to directly affect cognitive resilience. This study scoped the literature using a single search engine and was intentionally a preliminary investigation about associations between environmental factors and *CF.* Further research has been published and this is a growing area of study relating to social and economic status and the importance of wellbeing as a phenomenon in older adults.

## Conclusion

The association between green spaces and cognitive health outcomes are inconsistent *but* suggestive for a beneficial role of green space exposure on cognitive functions. This may be due to the opportunity for exercise and engagement in social activities or the experience of living in places that include cared for natural environments. Urban environments can be less safe and perceived as more limiting and isolating by older populations. Nature provokes feelings of renewal, restoration, and spiritual connectedness but this may not translate directly into cognitive resilience.

Health promotion using green space as a means of offering an evidence-based therapeutic milieu for older people may contribute to reducing cognitive frailty by increasing brain health. Cognitive frailty is an important focus for public health planning and while physiological mechanisms may be important the community environment and in particular, access to nature and green spaces is sufficiently associated with wellbeing and cognitive resilience to make it a priority for urban planning.

## Author contributions

SFD: Conceptualization, Formal analysis, Methodology, Supervision, Writing – original draft, Writing – review & editing. CB: Data curation, Formal analysis, Investigation, Project administration, Writing – review & editing. LN: Validation, Writing – review & editing. CD: Validation, Writing – review & editing.
